# MHD Two-Fluid Flow and Heat Transfer between Two Inclined Parallel Plates in a Rotating System

**DOI:** 10.1155/2014/256898

**Published:** 2014-12-16

**Authors:** P. Sri Ramachandra Murty, G. Balaji Prakash

**Affiliations:** Department of Mathematics, GIT, GITAM University, Visakhapatnam 530 045, India

## Abstract

Two-phase magnetohydrodynamic convective flow of electrically conducting fluid through an inclined channel is studied under the action of a constant transverse magnetic field in a rotating system. The fluids in the two phases are steady, incompressible, laminar, immiscible, and electrically conducting, having different densities, viscosities, and thermal and electrical conductivities. The transport properties of both the fluids are assumed constant. The bounding infinite inclined parallel plates are maintained at different constant temperatures, making an angle ϕ with the horizontal. Approximate solutions for velocity and temperature distributions are obtained by using a straightforward regular perturbation technique. An in-depth study has been done on the effects of rotation parameter, Hartmann number, inclination angle, the ratio of electrical conductivities, and viscosities of two fluids on the flow. It is observed that the effect of increasing rotation is to decrease the primary velocity. Further it is noticed that as the rotation increases, the secondary velocity increases for smaller rotation, while for larger rotation it decreases. It is also found that the temperature distribution decreases as the rotation increases.

## 1. Introduction

The study of convective Hartmann flow and heat transfer between two parallel plates is receiving considerable interest in the current literature. Shail [1] studied the problem of Hartmann flow of a conducting fluid in a horizontal channel of insulated plates with a layer of nonconducting fluid overlying a conducting fluid in a two-fluid flow. Rudraiah et al. [2] and Umavathi [3] have done a detailed analysis on free and forced convective heat transfer to an electrically conducting fluid in a channel. Lohrasbi and Sahai [4], Malashetty and Leela [5, 6], and Raju and Murty [7] have studied the Hartmann flow characteristics of two fluids in a horizontal channel. Seth et al. [8] have presented the detailed analysis on Hartmann flow in a rotating system in the presence of inclined magnetic field with Hall effects. Chauhan and Rastogi [9, 10] have considered Hall current and heat transfer effects on MHD flow and MHD Couette flow in a channel partially filled with a porous medium in a rotating system.

Numerous publications dealing with both the experimental and the theoretical aspects of the two-phase flow systems with or without considering the heat transfer problems associated with MHD power generators, MHD devices, and thermonuclear power generations have appeared in the literature. Abdul Mateen [11, 12] has studied the magnetohydrodynamic flow and transient magnetohydrodynamic flow of two immiscible fluids through a horizontal channel. Raju and Nagavalli [13] have studied the MHD two-layered unsteady flow and heat transfer through a horizontal channel in the presence of applied magnetic and electric fields in a rotating system. Recently, Murty and Linga Raju [14] have investigated magnetohydrodynamic two-phase flow and heat transfer between two parallel porous walls in a rotating system.

Basically, the inclined geometry has enormous applications in the heat transfer technology like solar collector. Though such problems with inclined geometry are close to realistic practical situations, much attention has not been given to handling them except the studies by Malashetty and Umavathi [15], Malashetty et al. [16], S. Daniel and Y. S. Daniel [17], and Murty and Prakash [18]. Therefore, in this paper, we have studied two-phase magnetohydrodynamic convective flow of electrically conducting fluid through an inclined channel under the action of a constant transverse magnetic field when rotated by an angular velocity about an axis perpendicular to the plates.

## 2. Mathematical Formulation

**Figure 1:**
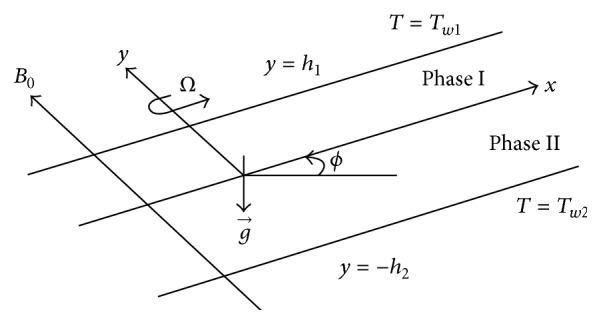
Physical configuration.

A steady laminar and fully developed two-phase magnetohydrodynamic convective flow driven by a common constant pressure gradient (−∂*p*/∂*x*) and temperature gradient Δ*T* = (*T*_*w*1_ − *T*_*w*2_) has been considered in the presence of a constant magnetic field applied transversely to the direction of the flow. The physical configuration is shown in Figure 1, which consists of two infinite inclined parallel plates maintained at different constant temperatures, extending in the *x* and *z* directions, making an angle *ϕ* with the horizontal. The regions 0 ≤ *y* ≤ *h*_1_ and −*h*_2_ ≤ *y* ≤ 0 are occupied by two different electrically conducting incompressible fluids having density *ρ*_*i*_, viscosity *μ*_*i*_, electrical conductivity *σ*_*i*_, and thermal conductivity *K*_*i*_. The whole system is rotated with an angular velocity Ω in a counterclockwise direction about *y*-axis perpendicular to the plates. The transport properties of both the fluids are assumed constant. The suffix *i*  (*i* = 1,2) represents the values for phases I and II, respectively.

With these assumptions, the governing equations of motion and energy for Boussinesq fluids as in Malashetty et al. [16] for both phases are

(1) μid2uidy2+ρigβiTi−Tw2sin⁡ϕ−σiB02ui =∂p∂x+2Ωρiwi,μid2widy2−σiB02wi=−2Ωρiui,d2Tidy2+μiKiduidy2+dwidy2 +σiB02ui2+wi2Ki=0,

where *u*_*i*_ and *w*_*i*_ are the primary and secondary velocity components along *x* and *z* directions, respectively, *T*_*i*_ is the temperature, *β*_*i*_ is the coefficient of thermal expansion, and *g* is the acceleration due to gravity. The fluid and the thermometric boundary conditions are unchanged by the addition of electromagnetic field. The no-slip condition requires that the velocity must be vanishing at the wall. In addition, the fluid velocity, shear stress, temperature, and heat flux must be continuous across the interface.

The boundary and interface conditions are

(2) u1h1=0,w1h1=0;u10=u20,w10=w20;u2−h2=0,w2−h2=0,μ1du1dy=μ2du2dy,μ1dw1dy=μ2dw2dy,at  y=0,T1h1=Tw1,T10=T20,T2−h2=Tw2,K1dT1dy=K2dT2dy  at  y=0.

In making these equations dimensionless, the following transformations are used:

(3) ui∗uiu−1,yi∗=yihi,θ=T−Tw2ΔT,m=μ1μ2,K=K1K2,h=h2h1,n=ρ2ρ1,b=β2β1,s=σ2σ1,Gr=gβ1h13ΔTν12,M=B0h1σ1μ1,Pr⁡=μ1CpK1,Ec=u−12CpΔT,Re=u−1h1ν1,P=h12μ1u−1∂p∂x,R2=Ωh12ν.

With the above nondimensional quantities, the governing equations (1) become

(4) d2uidy2+GrReAsin⁡ϕθi−BM2ui=CP+2R2wi,d2widy2−BM2wi=−2R2ui,d2θidy2+PrEc Dduidy2+dwidy2+PrEc FM2ui2+wi2=0,

where *A* = *bmnh* ^2^,  *B* = *msh* ^2^,  *C* = *mh* ^2^,  *D* = (*K*/*m*),  *F* = *Kh* ^2^ *s* , and *A*, *B*, *C*, *D*, and *F* are all equal to 1 for phase I.

The nondimensional forms of the boundary and interface conditions (2) become

(5) u110,w110;u10u20,w10w20;u2−10,w2−10,du1dy1mhdu2dy,dw1dy1mhdw2dy  at  y=0,θ111,θ10θ20,θ2−10,dθ1dy1Khdθ2dy  at  y=0.

The asterisks have been dropped for simplicity. Further writing *q*_1_ = *u*_1_ + *iw*_1_ and *q*_2_ = *u*_2_ + *iw*_2_, (4) can be written in complex form as

(6) d2qidy2+GrReAsin⁡ϕθi−BM2qi=CP−2iR2qi,

(7) d2θidy2+PrEc Ddqidydq−idy+PrEc FM2qiq−i=0

which are to be solved subject to the boundary and interface conditions:

(8) q110,q10q20,q2−10,dq1dy1mhdq2dy  at  y=0,θ111,θ10θ20,θ2−10,dθ1dy1Khdθ2dy  at  y=0.

## 3. Solutions

The governing equations of momentum (6) along with the energy equations (7) are to be solved subject to the boundary and interface conditions (8) for the velocity and temperature distributions. Here, we consider the Eckert number very small. Hence, the product PrEc ( = *ε*) is very small and can be exploited to use the regular perturbation method. The solutions are assumed in the form

(9) $$\begin{array}{c} \left(q_{i},\theta_{i}\right)=\left(q_{i0},\theta_{i0}\right)+\varepsilon \left(q_{i1},\theta_{i1}\right)+\cdots , \end{array}$$

where *q*_*i*0_, *θ*_*i*0_ are solutions for the case *ε* equal to zero. *q*_*i*1_, *θ*_*i*1_ are perturbed quantities relating to *q*_*i*0_, *θ*_*i*0_, respectively. Substituting the above solution in (6) and (7) and equating the coefficients of similar powers of *ε* to zero, we get the zeroth and the first order equations as follows.

Zeroth order equations:

(10) d2qi0dy2+GrReAsin⁡ϕθi0−BM2qi0 =CP−2iR2qi0,d2θi0dy2=0.

First order equations:

(11) d2qi1dy2+GrReAsin⁡ϕθi1−BM2qi1=−2iR2qi1,d2θi1dy2+Ddqi0dydq−i0dy+FM2qi0q−i0=0.

The corresponding Boundary conditions (8) reduce to

(12) q1010,q100q200,q20−10,dq10dy1mhdq20dy  at  y=0,θ1011,θ100θ200,θ20−10,dθ10dy1Khdθ20dy  at  y=0,

(13) q1110,q110q210,q21−10,dq11dy1mhdq21dy  at  y=0,θ1110,θ110θ210,θ21−10,dθ11dy1Khdθ21dy  at  y=0.

We observe that (10) and (11) are linear and coupled and therefore can be solved exactly. Here, we consider *q*_10_ = *u*_10_ + *iw*_10_,  *q*_20_ = *u*_20_ + *iw*_20_,  *q*_11_ = *u*_11_ + *iw*_11_, and *q*_21_ = *u*_21_ + *iw*_21_.

Solutions of the zeroth order equations (10) using boundary conditions (12) are

(14) $$\begin{array}{c} \theta_{10}=\frac{\left(y+Kh\right)}{\left(1+Kh\right)},\\ \theta_{20}=\frac{\left(1+y\right)Kh}{\left(1+Kh\right)}, \end{array}$$

(15) u10=c1eA5y+c2e−A5·ycos⁡A6y+A14+A12y,w10=−c1eA5y−c2e−A5·ysinA6y−A15−A13y,u20=c3eA23y+c4e−A23·ycos⁡A24y+A30+A32y,w20=−c3eA23y−c4e−A23·ysin⁡A24y−A31−A33y.

Solutions of the first order equations (11) using boundary conditions (13) are

(16) θ11=c5y+c6+B56e2A5y+B57cos⁡⁡2A6yhhh+B58e−2A5y+B132eA5ysin⁡A6y+B98y2hhh+B133eA5ycos⁡⁡A6y+B134e−A5ysin⁡A6yhhh+B135e−A5ycos⁡⁡A6y+B136eA5yycos⁡⁡A6yhhh+B137eA5yysin⁡A6y+B138e−A5yycos⁡⁡A6yhhh+B139e−A5yysin⁡A6y+B96y3+B97y4 +iB99sin⁡2A6y+B140eA5ysin⁡A6yhhhhh+B141eA5ycos⁡⁡A6y+B142e−A5ysin⁡A6yhhhhh+B143e−A5ycos⁡⁡A6y+B144eA5yycos⁡A6yhhhhh+B145eA5yysin⁡A6y+B146e−A5yycos⁡A6yhhhhh+B147e−A5yysin⁡A6y,θ21=c7y+c8+E67e2A23y+E68cos⁡⁡2A24yhhh+E69e−2A23y+E135eA23ysin⁡A24yhhh+E136eA23ycos⁡⁡A24y+E137e−A23ysin⁡A24yhhh+E138e−A23ycos⁡⁡A24y−E107y4−E108y3hhh+E139eA23yycos⁡⁡A24y+E140eA23yysin⁡A24yhhh+E141e−A23yycos⁡⁡A24y−E109y2hhh+E142e−A23yysin⁡A24y +iE110sin⁡2A24y+E143eA23ysin⁡A24yhhhhh+E144eA23ycos⁡⁡A24y+E145e−A23ysin⁡A24yhhhhh+E146e−A23ycos⁡A24y+E147eA23yycos⁡⁡A24yhhhhh+E148eA23yysin⁡A24y+E149e−A23yycos⁡⁡A24yhhhhh+E150e−A23yysin⁡A24y,u11=c9eA5y+c10e−A5ycos⁡⁡A6y +F95e2A5y+F96e−2A5y−F97cos⁡⁡2A6y +F98sin⁡2A6y+F99eA5yysin⁡A6y +F100eA5yycos⁡⁡A6y−F101e−A5yycos⁡⁡A6y −F107y4−F108y3+F102e−A5yysin⁡A6y +F103eA5yy2sin⁡A6y+F104eA5yy2cos⁡⁡A6y −F109y2−F110y+F105e−A5yy2sin⁡A6y −F106e−A5yy2cos⁡⁡A6y−F111,w11=c10e−A5y−c9eA5ysin⁡A6y −F112e2A5y−F113e−2A5y−F114cos⁡⁡2A6y −F115sin⁡2A6y+F116eA5yycos⁡⁡A6y +F117eA5yysin⁡A6y−F125y3−F126y2 −F118e−A5yycos⁡⁡A6y+F119e−A5yysin⁡A6y +F120eA5yy2sin⁡A6y−F127y−F128 +F121eA5yy2cos⁡⁡A6y+F122e−A5yy2sin⁡A6y −F123e−A5yy2cos⁡⁡A6y−F124y4,u21=c11eA23y+c12e−A23ycos⁡⁡A24y +G96e2A23y+G97e−2A23y+G98cos⁡⁡2A24y +G112−G99sin⁡2A24y+G100eA23yysin⁡A24y +G101eA23yycos⁡⁡A24y+G108y4+G109y3 +G102e−A23yycos⁡⁡A24y+G103e−A23yysin⁡A24y +G104eA23yy2sin⁡A24y+G110y2 +G105eA23yy2cos⁡⁡A24y+G106e−A23yy2cos⁡⁡A24y +G107e−A23yy2sin⁡A24y+G111y,w21=c12e−A23y−c11eA23ysin⁡A24y −G113e2A23y−G114e−2A23y+G115cos⁡⁡2A24y +G129+G116sin⁡2A24y+G117eA23yysin⁡A24y +G118eA23yycos⁡⁡A24y+G128y+G127y2 +G119e−A23yycos⁡⁡A24y+G120e−A23yysin⁡A24y +G121eA23yy2sin⁡A24y+G126y3 +G122eA23yy2cos⁡⁡A24y+G123e−A23yy2sin⁡A24y +G124e−A23yy2cos⁡⁡A24+G125y4.

The constants appearing in (15) and (16) are not given for the sake of brevity. Since the problem contains too many nondimensional parameters, for the sake of conciseness, we fix *P* = −5.0, *b* = 1.0, *Re* = 5.0, *n* = 1.5, and *K* = 1.0. In the figures, all the other parameters except the varying one are chosen from the set (*M*, Gr, *ϕ*, *m*, *h*, *s*, *R*) = (2.0, 5.0, 30°, 0.5, 1.0, 2.0, 2.0).

## 4. Results and Discussion

Two-phase magnetohydrodynamic convective flow between two infinite inclined parallel plates in a rotating system is studied analytically. The resulting differential equations are solved using perturbation method to obtain approximate solutions for temperature distribution and primary and secondary velocity distributions. Here, we note that when *R* = 0, that is, in the absence of rotation, these results are in agreement with that of Malashetty et al. [16].

**Figure 2:**
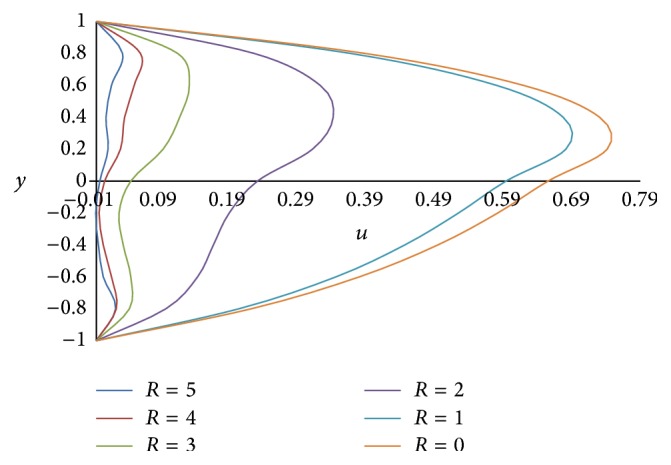
Primary velocity profiles for different values of rotation parameter *R*.

**Figure 3:**
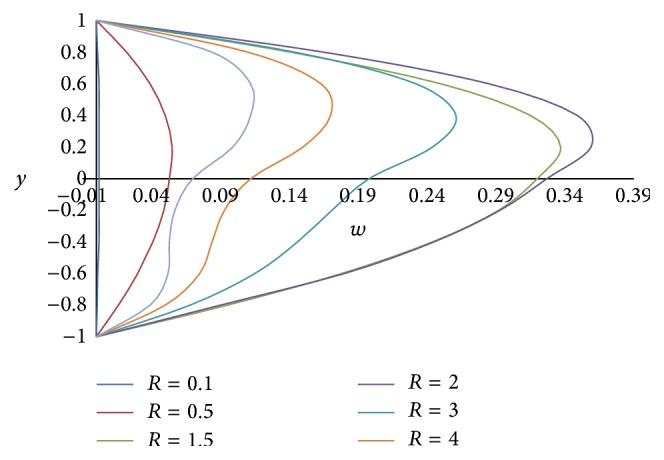
Secondary velocity profiles for different values of rotation parameter *R*.

The effect of rotation parameter *R* on primary velocity *u* and secondary velocity *w* is shown in Figures 2 and 3, respectively. From Figure 2, it is observed that the primary velocity *u* decreases with the increase in the rotation parameter. The rotation parameter *R* defines the relative magnitude of the Coriolis force and the viscous force in the regime. As the high magnitude Coriolis forces oppose the buoyancy force, the velocity will be decreased. From Figure 3, it is concluded that as the rotation parameter *R* increases in (0, 2), the secondary velocity *w* also increases but outside this range as *R* increases, it decreases. Therefore, by increasing the rotation parameter *R*, the secondary flow becomes oscillatory.

**Figure 4:**
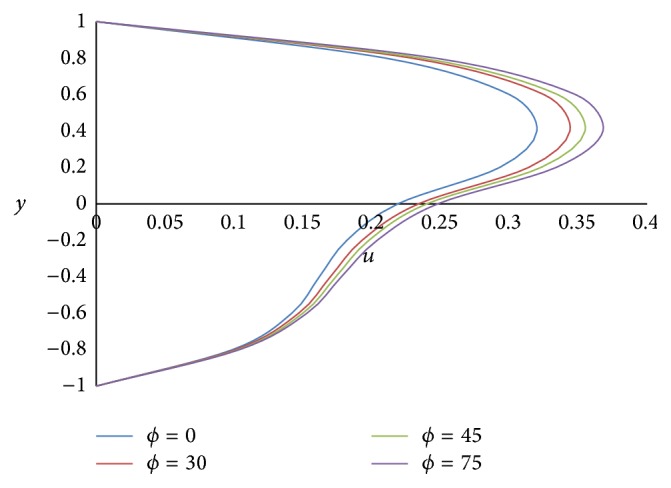
Primary velocity profiles for different values of inclination angle *ϕ*.

**Figure 5:**
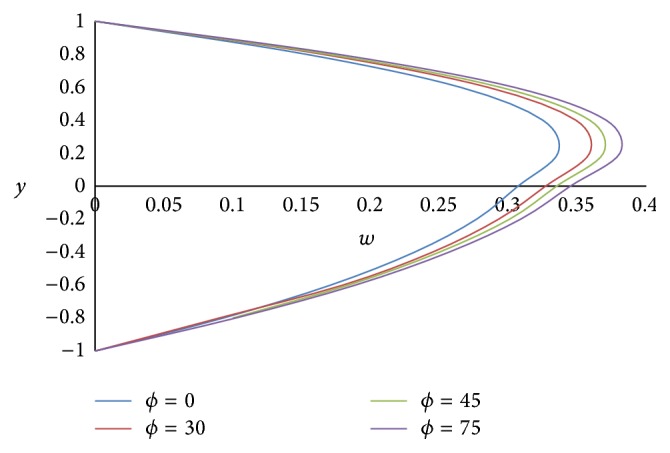
Secondary velocity profiles for different values of inclination angle *ϕ*.

**Figure 6:**
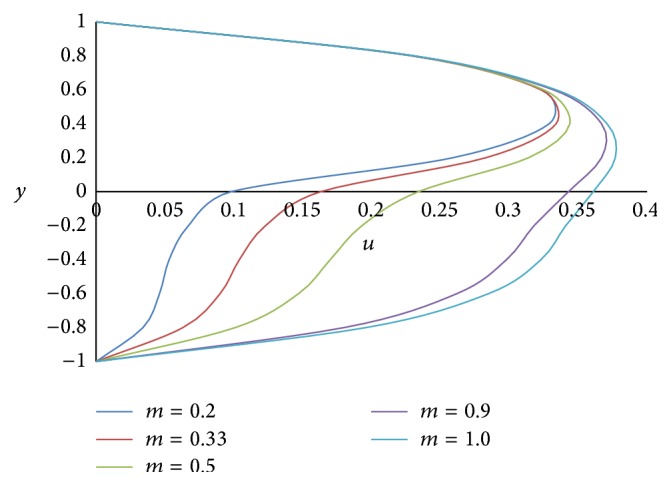
Primary velocity profiles for different values of ratio of viscosities *m*.

**Figure 7:**
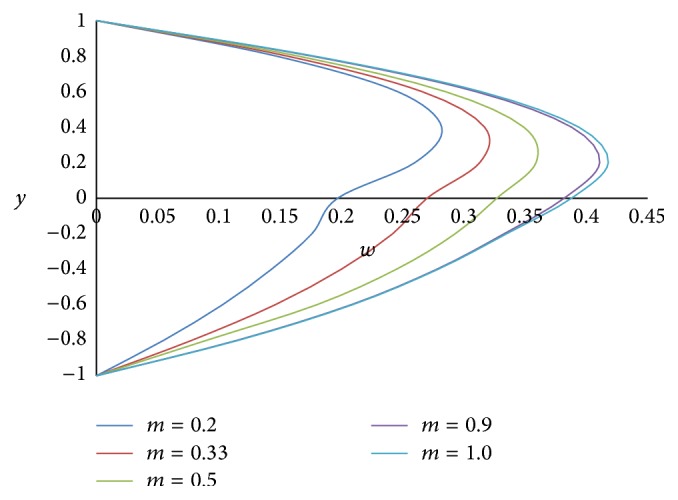
Secondary velocity profiles for different values of ratio of viscosities *m*.

Figures 4 and 5 show the effect of the angle of inclination *ϕ* on primary velocity *u* and secondary velocity *w*, respectively. As the angle of inclination *ϕ* increases, both the primary and secondary velocities increase because the magnitude of the buoyancy force increases with increase in the inclination angle. The effect of the ratio of the viscosities *m* on primary and secondary velocities is shown in Figures 6 and 7, respectively. The smaller the value of the viscosity of the fluid in the lower phase compared to the fluid in the upper phase, the larger the primary and secondary flow fields.

**Figure 8:**
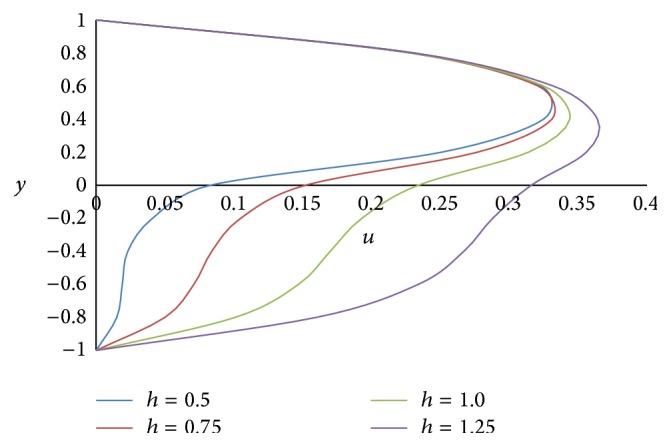
Primary velocity profiles for different values of ratio of heights *h*.

**Figure 9:**
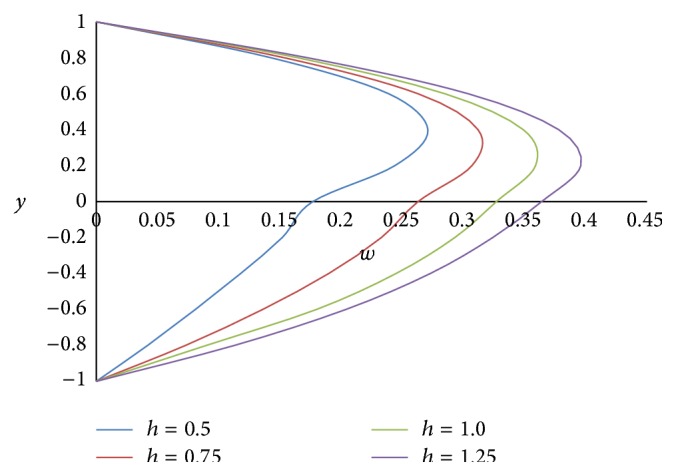
Secondary velocity profiles for different values of ratio of heights *h*.

**Figure 10:**
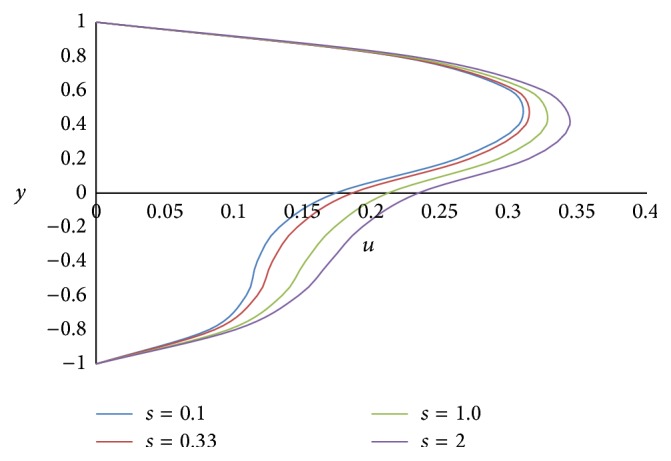
Primary velocity profiles for different values of ratio of electrical conductivities *s*.

**Figure 11:**
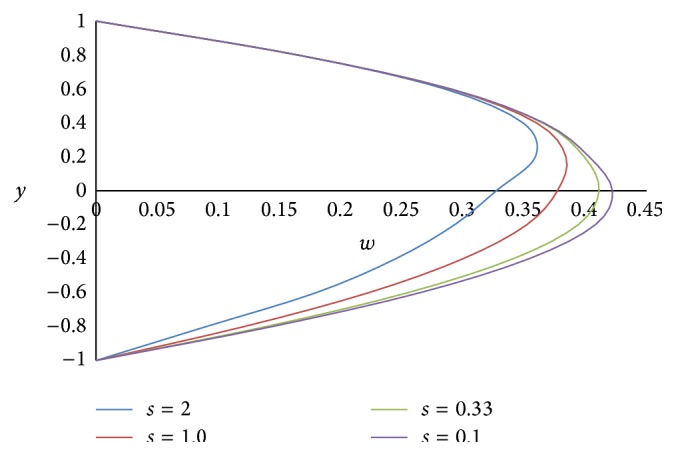
Secondary velocity profiles for different values of ratio of electrical conductivities *s*.

Figures 8 and 9 represent the effect of the ratio of heights *h* on primary and secondary velocities, respectively. The smaller the height of the upper phase compared to the lower phase, the larger the primary as well as secondary flow fields. The effect of the ratio of the electrical conductivity *s* on primary velocity *u* and secondary velocity *w* is shown in Figures 10 and 11, respectively. We have observed that as the ratio of the electrical conductivity *s* increases, the primary velocity *u* increases but the secondary velocity *w* decreases.

**Figure 12:**
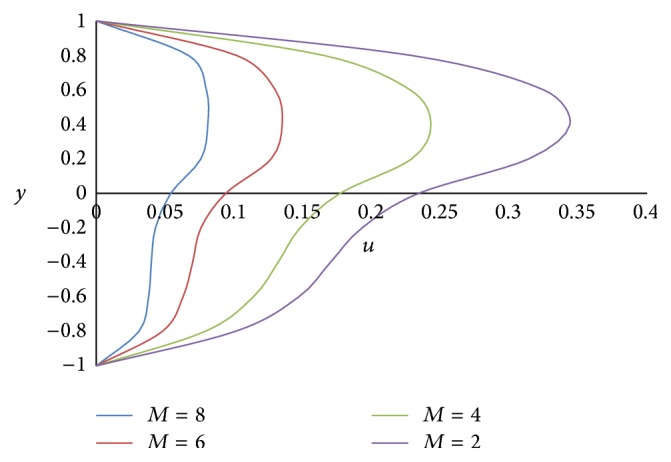
Primary velocity profiles for different values of Hartmann number *M*.

**Figure 13:**
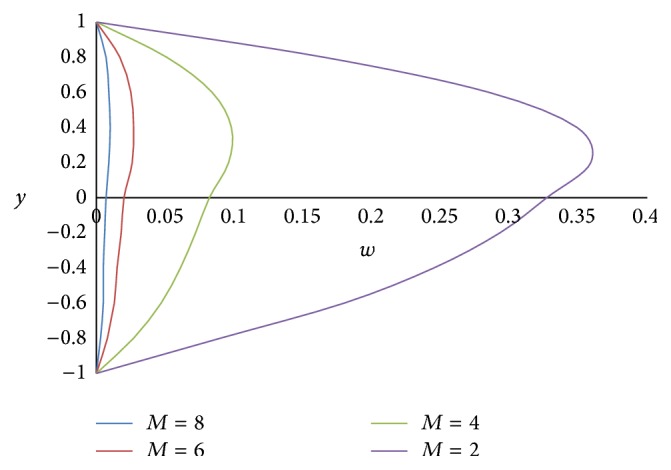
Secondary velocity profiles for different values of Hartmann number *M*.

**Figure 14:**
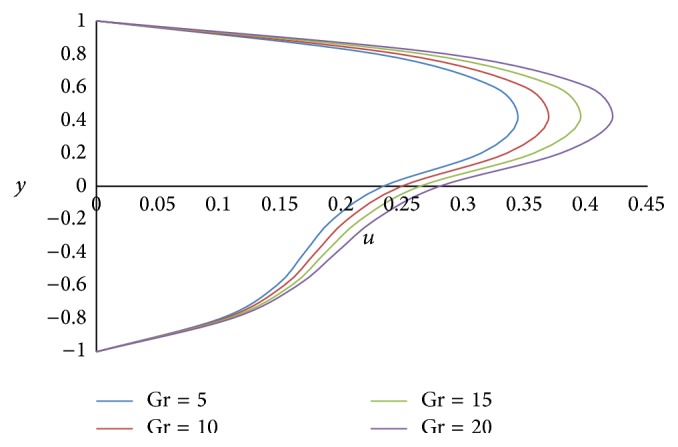
Primary velocity profiles for different values of Grashof number Gr.

**Figure 15:**
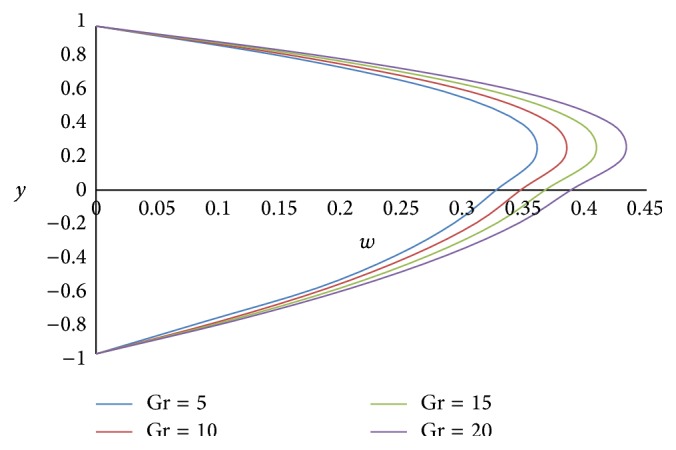
Secondary velocity profiles for different values of Grashof number Gr.

Figures 12 and 13 show the effect of Hartmann number *M* on primary and secondary velocities, respectively. The effect of increasing Hartmann number *M* is to decrease both the primary and secondary velocities. This is because an increase in applied magnetic field strength causes greater interaction between the fluid motion and the magnetic field, therefore, an increase in the Lorentz force. Since this force opposes the buoyancy force, both the velocities will be decreased. The effect of Grashof number Gr on primary and secondary velocities is shown in Figures 14 and 15, respectively. We find that an increase in the value of Grashof number Gr increases both the primary and secondary velocities.

**Figure 16:**
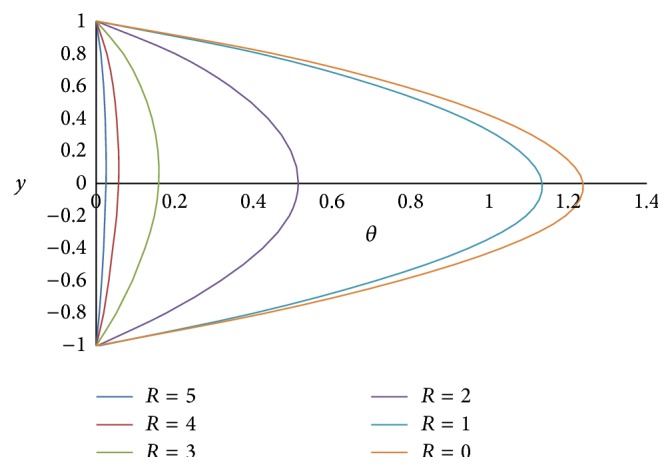
Temperature profiles for different values of rotation parameter *R*.

The effect of rotation parameter on temperature *θ* can be seen in Figure 16. From the figure, it is evident that the temperature decreases with the increase in the rotation parameter *R*. The rotation parameter *R*  (*R* ^2^ = *Ωh*_*i*_^2^/*ν*) defines the relative magnitude of the Coriolis force and the viscous force in the regime. As the high magnitude Coriolis forces oppose the buoyancy force, the velocity will be decreased leading to a reduction in the viscous and Joule dissipation and so to a reduction in the temperature.

**Figure 17:**
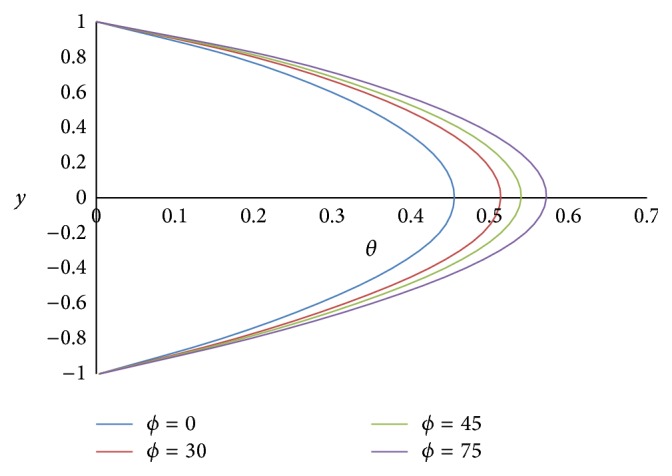
Temperature profiles for different values of inclination angle *ϕ*.

**Figure 18:**
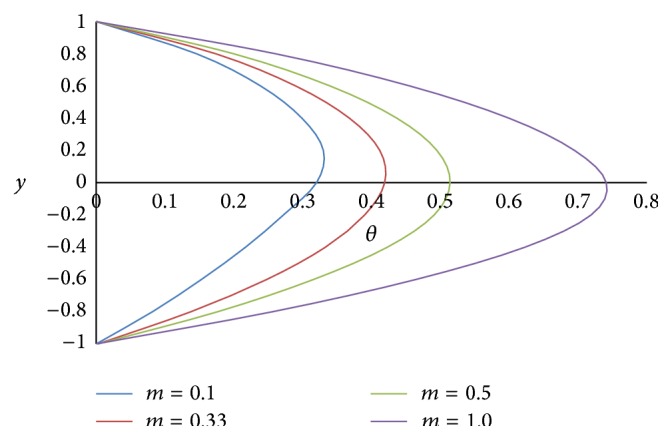
Temperature profiles for different values of ratio of viscosities *m*.

**Figure 19:**
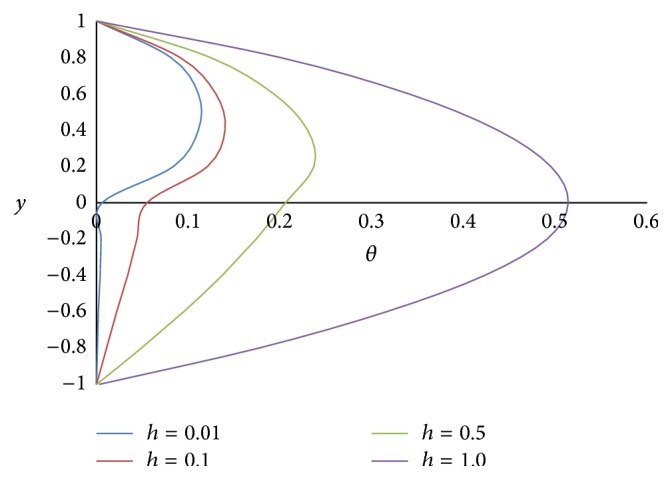
Temperature profiles for different values of ratio of heights *h*.

**Figure 20:**
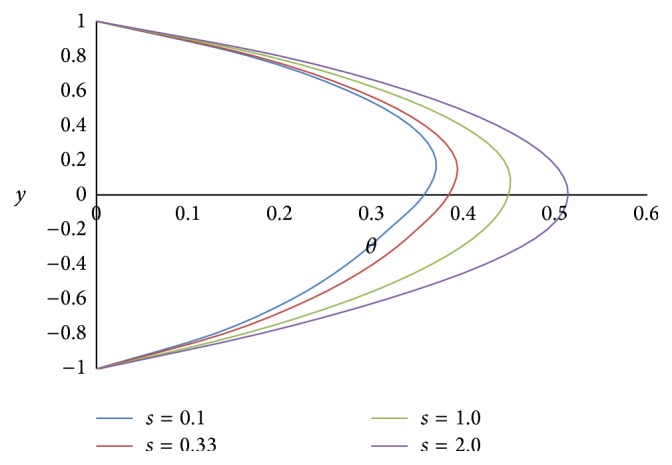
Temperature profiles for different values of ratio of electrical conductivities *s*.

Figure 17 shows the effect of the angle of inclination *ϕ* on temperature *θ*. As the angle of inclination *ϕ* increases, the temperature *θ* also increases because the magnitude of the buoyancy force increases with the increase in the inclination angle. The effect of the ratio of viscosities *m* on the temperature *θ* is shown in Figure 18. From the figure, it is observed that the less viscous fluid in the lower phase adds the heat transfer.  Figure 19 exhibits the effect of the ratio of heights *h* on the temperature *θ*. From the figure, it is noticed that the smaller the height of the upper phase compared to the lower phase, the larger the magnitude of the temperature.  Figure 20 shows the effect of the ratio of electrical conductivities *s* on the temperature *θ*. From the figure, it is concluded that as the ratio of electrical conductivities *s* increases, the temperature also increases.

**Figure 21:**
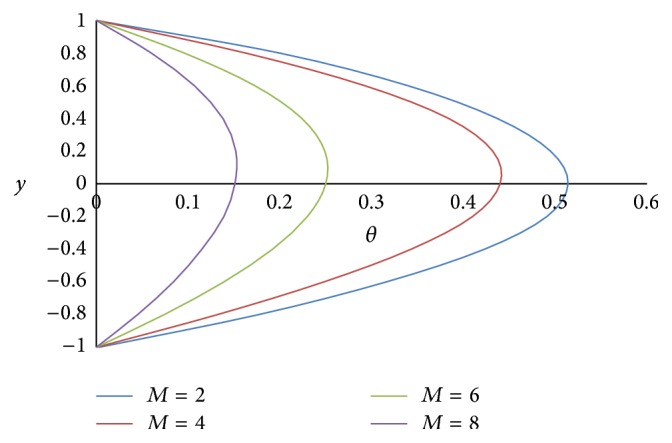
Temperature profiles for different values of Hartmann number *M*.

**Figure 22:**
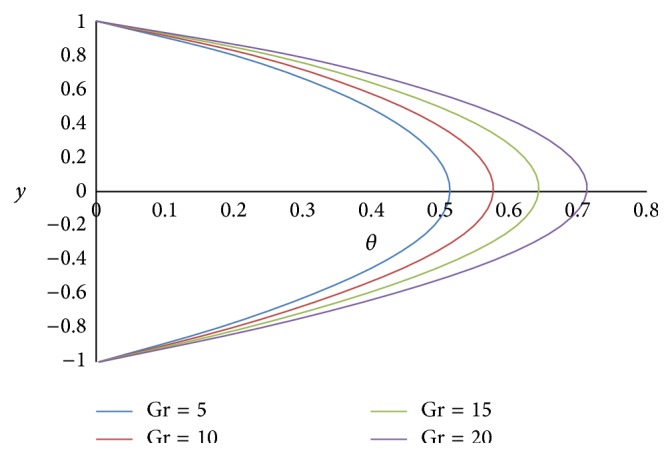
Temperature profiles for different values of Grashof number Gr.

Figure 21 represents the effect of Hartmann number *M* on the temperature *θ*. From the figure, it is clear that the effect of increasing *M* is to decrease the temperature. This is because an increase in the applied magnetic field strength causes greater interaction between the fluid motion and the magnetic field, therefore, an increase in the Lorentz force. Since this force opposes the buoyancy force, the temperature will be decreased. The effect of Grashof number Gr on temperature *θ* is shown in Figure 22; we observe from this figure that an increase in the value of Grashof number Gr increases the temperature *θ*.

## Nomenclature

*B*_0_: Magnetic field strength
*b*: Ratio of the coefficients of thermal expansion (*β*_2_/*β*_1_)
*C*_*p*_: Specific heat at constant pressure
Ec: Eckert number $\left[{\left({\overset{-}{u}}_{1}\right)}^{2}/\left(C_{p}\Delta T\right)\right]$
*g*: Acceleration due to gravity
*h*: Ratio of the heights of the two phases (*h*_2_/*h*_1_)
Gr: Grashof number [*gβ*_1_ *h*_1_^3^Δ*T*/*ν*_1_^2^]
*K*: Ratio of the thermal conductivities (*K*_1_/*K*_2_)
*K*_1_, *K*_2_: Thermal conductivities of phases I and II, respectively
*M*: Hartmann number $\left[B_{0}h_{1}\sqrt{\left(\sigma_{1}/\mu_{1}\right)}\right]$
*m*: Ratio of the viscosities (*μ*_1_/*μ*_2_)
*n*: Ratio of the densities (*ρ*_2_/*ρ*_1_)
*P*: Nondimensional pressure gradient $\left[h_{1}^{2}\left(\partial p/\partial x\right)/\mu_{1}{\overset{-}{u}}_{1}\right]$
*Pr*⁡: Prandtl number [(*μ*_1_ *C*_*p*_/*K*_1_)]
*Re*: Reynolds number $\left[\left({\overset{-}{u}}_{1}h_{1}/\nu_{1}\right)\right]$
*R*: Rotation parameter $\left[h_{1}\sqrt{\Omega /\nu }\right]$
*s*: Ratio of the electrical conductivities (*σ*_2_/*σ*_1_)
*T*: Temperature
*T*_*w*1_, *T*_*w*2_: Temperature of the boundaries
*u*: Primary velocity
*w*: Secondary velocity
${\overset{-}{u}}_{1}$: Average velocity
*x*, *y*, *z*: Space coordinates.

### Greek Symbols

*β*: Coefficient of thermal expansion
*σ*: Electrical conductivity
*ϕ*: Angle of inclination
*ρ*: Density
*ν*: Kinematic viscosity
*μ*: Viscosity
*ε*: Product of Prandtl number and Eckert number (*Pr*⁡·Ec)
Δ*T*: Difference in temperature [*T*_*w*1_ − *T*_*w*2_]
*θ*: Nondimensional temperature [(*T* − *T*_*w*2_)/Δ*T*]
*Ω*: Angular velocity.

### Subscript

*i*: Value for phase.
